# Progress in Bacteriology

**Published:** 1901-11-02

**Authors:** 


### PROGRESS IN BACTERIOLOGY.

(*Continued from page* 35.)

**The Filiform Baccillus and Cancer of the Stomach.**—Ehret[12] states that the normal stomach always contains yeasts, sarcinæ and cocci, and that in diseased conditions leading to achlorhydria and stasis the fermentative changes are due to the multiplication of these species, and not to the introduction of new varieties. Various forms of dyspepsia are due to multiplication of one or other species. He asserts that a bacillus which he calls the filiform bacillus is always to be found in the stomach contents in cases of cancer of this organ, that the organisms are present in very large numbers, and that it is possible to diagnose the disease from their presence at a very early date. The filiform bacillus is a long thin thread-like organism, which may form large tangled masses in the vomited material. It grows on glycerine agar, forming in the second day minute, white, dry colonies of almost transparent clearness. It forms a thick flocculent precipitate in broth, but does not grow on gelatine. In some media it forms lactic acid. This bacillus is not found in the normal stomach, and its presence in enormous numbers in stomach contents is in Ehret's opinion absolutely diagnostic of malignant disease, even though no other symptoms be present. Out of 29 cases in the Strasbourg clinic in which this organism was present, 25 were shown, either by subsequent operative procedure or at post-mortem examination, to have cancer of the stomach. Boas was the first to draw attention to this organism, and Oppler, Schlesinger and Kaufmann have confirmed his results. Hammeter[13] describes the bacillus as a long rod-shaped organism, non-motile and usually thicker at one end than the other. He found it present in 53 out of 56 cases of cancer of the stomach, but does not regard its presence as being pathognomonic of this condition. It is not present early in the disease, and has about the same significance as the finding of lactic acid.

**Tubercle Infection of Urine.**—Foulerton and Hillier[14] have examined the urine for tubercle bacilli in 24 cases of pulmonary phthisis. Early in their experience they decided that mere microscopical examination of the sediment was useless, and in 19 subsequent cases injection of the sediment obtained by centrifugalisation was made into guinea-pigs. In nine instances tubercle bacilli were found to be present. Of these nine cases six were proved by post-mortem examination to be free from tubercular disease of the kidney, whilst one showed tubercular foci of disease, and the remainder recovered. Of the cases in which no tubercle bacilli were found only one showed albuminuria, whilst in the infected series seven gave evidence of a trace, but in none was there histological evidence of nephritis. The authors draw the following conclusions :—A general blood infection is not uncommon in phthisis, but the few bacilli present in the blood are speedily excreted by the kidneys, seldom giving rise to infection of these organs.

**Cystitis.**[15]—Over 20 different organisms have been isolated from pathological urines. Amongst these the earliest to be discovered were the micrococcus ureæ by Pasteur, the bacterium pyogenes of Albairan and Halle, and bacteria described by Bumm, Michaelis, Bouchard and Clado, which were all probably polymorphic varieties of the bacillus coli. Other varieties are the streptococcus pyogenes, staphylococcus albus, aureus, and citreus, uro-bacillus liquefaciens septicus (proteus), gonococcus, and tubercle bacillus. The bladder may be infected by a posterior urethritis, or when there is incontinence and the sphincter is relaxed direct growth along the saturated urethra may take place. In acute and chronic enteritis and colitis bacteria enter the blood and may so infect the urine. Posner and Lewin showed that this happened in animals whose intestines had been ligatured. The kidneys are generally the site of the original infection, but the bacteria may also be initially deposited in the bladder wall. Diffuse inflammation or abscess of neighbouring structures may lead to bladder infection. The bacillus coli may in some cases be present in enormous numbers without giving rise to any other symptoms than a slight mousey odour. In other cases a typical cystitis is developed. The explanation of this fact is not known. Pure cultures of gonococci injected into the bladder do not give rise to cystitis. Tuberculous cystitis is generally secondary. The irritation arising from trauma, foreign bodies, or residual urine necessarily precedes the bacterial infection. Vaseline and fat harbour bacteria, and are bad lubricants for catheters. Lewis[16] states that typhoid bacilluria always gives rise to pus in urine, and may, especially if associated with bacillus coli, give rise to symptoms of cystitis. He only found typhoid bacilluria once in 45 cases of enteric fever. Petruschky found it in six per cent. of cases, Richardson and Horton Smith in 25 per cent. It is seldom present before the third week.

**Cerebro-Spinal Meningitis.**—Hunter and Nuthall[17] examined ten cases of cerebro-spinal meningitis by means of lumbar puncture. Nine of these cases showed cocci, some of which resembled the micrococcus intracellularis of Weichselbaum, and others that of posterior basic meningitis. The authors believe that there is no true distinction between these two organisms, but that they are varying types of the same bacillus, and that the so-called basic meningitis is simply a sporadic form of cerebro-spinal meningitis. Weichselbaum's diplococcus is distinguished by the following features: it does not stain by Gram's method; the growth is feeble on agar, fine and transparent, and growth on blood serum and potatoe is scanty. Neither variety is pathogenic to animals. In only four cases was the organism found in pure culture, the B. influenzæ was present in three, the B. meningitidis (Centanni [18]) in two, cocci in two, and tubercle in one. In a further communication they point out that this organism is distinct from the pneumococcus which it closely resembles, and that recently a spinal meningitis has been produced in goats by inoculation with pure cultures. In the discussion which followed the paper Foulerton expressed a doubt as to whether any diplococcus which was not decolorised by Gram's method could rightly be described as the diplococcus intracellularis. Lazarus Barlow [19] points out that the name "posterior basic meningitis" is not descriptive of a distinct bacteriological entity. He reports two cases in which the symptoms were typically those of the posterior basic character, and in which exudation was found over the base after death, and in both of which pneumococci only were found.

**Neisser's Stain.**—Beaton, Caiger, and Pakes [20] have tested the value of this stain in the diagnosis of diphtheria. They recommend that the films be first stained for two minutes in the methylene blue solution, and after washing in water, stained for a further two minutes in the watery solution of vesuvin. Formerly it was held that the stains should only be used for a few seconds. They claim that in some cases where diagnosis is difficult with the methylene blue method, Neisser's method gives ready means of diagnosis. The great advantage of the method is that diagnosis may be made direct from the films without having to wait for cultures to be incubated. This was possible in 18 per cent. of all their cases, and in 37·5 per cent. of the cases proved to be diphtheria. These results are confirmed by Cammidge,[21] who was able to detect diphtheria bacilli in direct films in 33 per cent. of cases. For culture medium he prefers solidified hydrocele fluid, and for staining 30 seconds in Neisser's solution, followed by half to one minute in bismark brown. Cultures may be examined in from six to eight hours.

[12] La Sem. Med., March 6. 1901. [13] Lancet, Sept. 28, 1901. [14] Brit. Med. Jour., Sept. 21, 1901. [15] Med. Rev. of Rev., Aug. 25. 1901. [16] Edin. Med. Jour., Sept, 1901. [17] Lancet, June 1. 1901. [18] Brit. Med. Jour., Sept. 21, 1901. [19] Ibid. [20] Ibid. [21] Brit. Med. Jour., Oct. 5, 1901.

